# Ticagrelor Induces Angiogenesis in Progenitor and Mature Endothelial Cells In Vitro: Investigation of the Possible Role of Adenosine

**DOI:** 10.3390/ijms252413343

**Published:** 2024-12-12

**Authors:** Sofia Sidiropoulou, Aikaterini Gatsiou, Kenny M. Hansson, Aikaterini N. Tsouka, Konstantinos Stellos, Alexandros D. Tselepis

**Affiliations:** 1 Atherothrombosis Research Centre/Laboratory of Biochemistry, Department of Chemistry, University of Ioannina, 451 10 Ioannina, Greece; sidiropoulous@hotmail.com (S.S.); tsoukakaterina@hotmail.gr (A.N.T.); 2 Cardiovascular Disease Prevention Hub, Faculty of Medical Sciences, Newcastle University, Newcastle Upon Tyne NE1 7RU, UK; aikaterini.gatsiou@ncl.ac.uk (A.G.); konstantinos.stellos@medma.uni-heidelberg.de (K.S.); 3 Bioscience Cardiovascular, Research and Early Development, Cardiovascular, Renal and Metabolism (CVRM), BioPharmaceuticals R&D, AstraZeneca, 431 50 Gothenburg, Sweden; kenny.m.hansson@astrazeneca.com; 4 Freeman Hospital, Newcastle Upon Tyne Hospitals NHS Foundation Trust, Newcastle Upon Tyne NE7 7DN, UK; 5 Department of Cardiology, University Hospital Mannheim, University of Heidelberg, 69117 Mannheim, Germany; 6 Department of Cardiovascular Research, Medical Faculty Mannheim, Heidelberg University, 69117 Mannheim, Germany

**Keywords:** adenosine, angiogenesis, endothelial progenitor cells, endothelial colony-forming cells, equilibrative nucleoside transporter-1, ticagrelor

## Abstract

Ticagrelor, a reversible platelet P2Y_12_ receptor antagonist, exerts various pleiotropic actions, some of which are at least partially mediated through adenosine. We studied the ticagrelor and adenosine effect on the angiogenic properties of progenitor CD34^+^-derived endothelial colony-forming cells (ECFCs). Angiogenesis studies were performed in vitro using capillary-like tube formation and spheroid-based angiogenesis assays. The effects of adenosine receptor antagonists, including DPCPX (A_1_ antagonist), SCH58621 (A_2A_ antagonist), MRS1706 (A_2B_ inverse agonist and antagonist), MRS1220 (A_3_ antagonist) and adenosine deaminase (ADA), were also investigated. Ticagrelor, adenosine, and their combination increased capillary-like tube formation and spheroid sprout formation by ECFCs in a dose-dependent manner. This effect was significantly reduced by SCH58621, MRS1706, and their combination, as well as by ADA. By contrast, DPCPX and MRS1220 did not exhibit any inhibitory effects. Similar results were obtained when mature human umbilical vein endothelial cells (HUVECs) were studied. These results show that ticagrelor stimulates angiogenesis by progenitor and mature endothelial cells in an adenosine-dependent pathway in which the adenosine receptors A_2A_ and A_2B_ play major roles. The significance of these results at the clinical level in patients with atherothrombotic events and treated with ticagrelor needs to be investigated.

## 1. Introduction

Endothelial progenitor cells (EPCs) are a multifaceted cell population that can differentiate to mature endothelial cells (ECs) when higher cells are reorganized or under conditions of oxidative stress, and they contribute to the formation of new blood vessels [1,2,3]. Several studies have demonstrated that EPCs are capable of self-renewal and differentiation into mature endothelial cells, contributing significantly to the regeneration of the vascular endothelium by re-endothelialization. This ability of EPCs remains of high interest for regenerative medicine [4,5,6]. A more recent study showed that the definition of EPCs as cells from circulating blood that promote new blood vessel formation is not sufficiently accurate in the era of precision medicine [7,8].

Depending on the method of isolation and culture, as well as the cell phenotype, several EPC subtypes have been described [9,10]. Among them, late EPCs, also known as late-outgrowth endothelial cells (OECs) or endothelial colony-forming cells (ECFCs), exhibit remarkable potential for proliferation and promoting angiogenesis [11]. Moreover, ECFCs display a greater ability to form a capillary tube structure that integrates into mature EC monolayers, as well as migration potential, leading to new therapeutic neovascularization strategies in the regeneration of ischemic cardiac tissues and blood vessels [12,13].

Ticagrelor is a reversible oral P2Y_12_ receptor antagonist administered in an active form; therefore, it does not require hepatic biotransformation [4,14]. In addition to its potent inhibitory effect on adenosine diphosphate (ADP)-induced platelet activation, ticagrelor exerts pleiotropic effects on various cell types, including ECs [15,16,17,18,19]. More specifically, ticagrelor moderates the progression of atherosclerosis through the adenosine-mediated attenuation of pro-inflammatory cytokines. Furthermore, ticagrelor can increase local interstitial adenosine levels, which activate downstream anti-inflammatory prostaglandins, eicosanoids, and AMPK. It also enables vasodilatory activity in infarcted vessels, protects against ischemia–reperfusion injury, and has antimicrobial effects [20]. One of the possible underlying mechanisms behind ticagrelor’s pleiotropic effects could be related to its ability to increase adenosine concentrations by blocking the equilibrative nucleoside transporter-1 (ENT-1) [21,22,23]. ENT1 and ENT2 are functionally the most relevant adenosine transporters in most cell types, as they have been shown to be nearly ubiquitously expressed in human tissues [24]. Adenosine is an endogenous nucleoside that functions as a signaling molecule through the activation of four G-protein coupled adenosine receptors (ARs), denoted as A_1_, A_2A_, A_2B_, and A_3_. The ARs are expressed in endothelial cells regulating every aspect of endothelial inflammatory processes [25,26]. Adenosine is a key regulator of angiogenesis by influencing the expression of pro-angiogenic factors, such as vascular endothelial growth factor (VEGF) [27,28]. Moreover, ENTs are polytopic integral membrane proteins that mediate the transport of nucleosides, nucleobases, and therapeutic analogs [29]. The ability of ENTs to regulate the flux of nucleosides, nucleobases, and nucleoside-derived therapeutics has far-reaching implications. Adenosine is of particular interest because of its wide-ranging effects on multiple organ systems by interacting with ARs, which govern cellular functions via the regulation of downstream heterotrimeric G-proteins. These observations have linked adenosine and ARs with the upregulation of angiogenesis processes.

In the present study, we evaluate the ability of ticagrelor to stimulate the pro-angiogenic properties of progenitor CD34^+^-derived ECFCs, and HUVECs in vitro, as well as the possible role of adenosine.

## 2. Results

### 2.1. Characterization of ECFCs

The ECFC characteristics were similar to those previously described by our group and others [8,30]. In brief, the ECFCs displayed typical cobblestone morphology, were double-positive for both DiI-ac-LDL uptake and FITC-(UEA)-1 lectin staining (see Supplementary Figure S1), as well as for the endothelial marker vWF. The ECFCs also expressed CD31, CD34, and KDR, as revealed by flow cytometry (see Supplementary Figure S2). 

### 2.2. Effects of Ticagrelor and Adenosine on Capillary-Like Tube and Spheroid Sprout Formation

**Figure 1:**
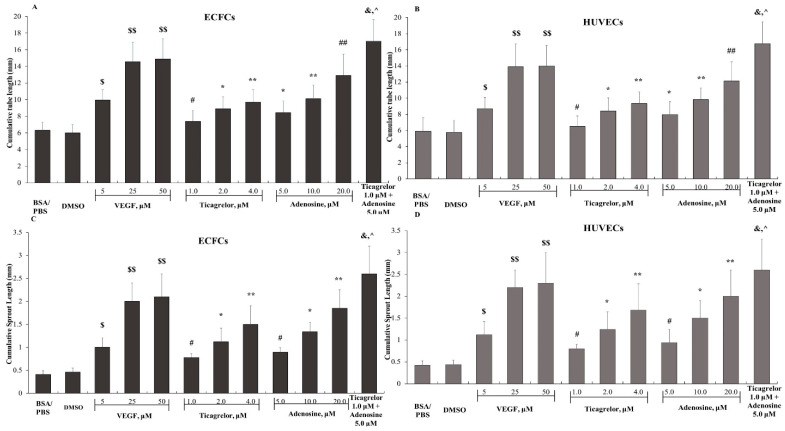
The dose-dependent effects of Vascular Endothelial Growth Factor (VEGF), ticagrelor, adenosine, and the combination of ticagrelor and adenosine on capillary-like tube formation by (**A**) Endothelial Colony-Forming Cells (ECFCs) and (**Β**) Human Umbilical Vein Endothelial Cells (HUVECs), and on spheroid sprout formation by (**C**) ECFCs and (**D**) HUVECs. The values represent the means ± SDs from at least 6 independent biological replicates each performed in 2 technical replicates. $ *p* < 0.02 and $$ *p* < 0.005 compared to BSA/PBS. # *p* < 0.05, * *p* < 0.02, ** *p* < 0.01, ## *p* < 0.005, and & *p* < 0.001 compared to DMSO. ^ *p* < 0.001 compared to 1.0 μM ticagrelor or 5.0 μM adenosine.

**Figure 2:**
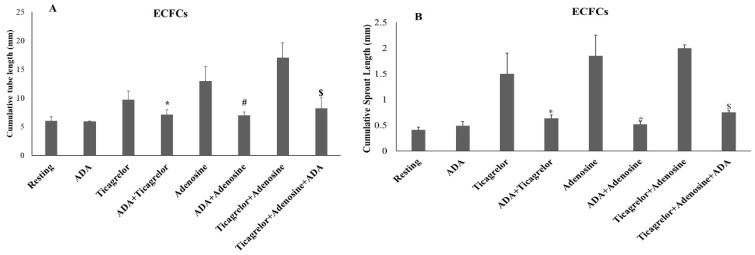
(**A**). The effects of Adenosine Deaminase (ADA) alone or in combination with ticagrelor or adenosine on capillary-like tube formation. (**B**). The effects of ADA (alone) or in combination with ticagrelor or adenosine on capillary-like tube formation on spheroid sprout formation. The values represent the means ± SDs from at least 6 independent biological replicates each performed in 2 technical replicates. (**A**). * *p* < 0.03 compared to ticagrelor alone, ^#^ *p* < 0.02 compared to adenosine, and ^$^ *p* < 0.005 compared to the combination of ticagrelor with adenosine. (**B**). * *p* < 0.02 compared to ticagrelor alone, ^#^ *p* < 0.01 compared to adenosine, and ^$^ *p* < 0.005 compared to the combination of ticagrelor with adenosine.

The cumulative length of the tube network formed by ECFCs in the presence of 0.1% *w*/*v* BSA/PBS was 6.33 ± 0.95 mm (Figure 1A). This value was not significantly altered in the experiments performed in the presence of 0.4% *v*/*v* DMSO. Ticagrelor significantly increased tube formation by ECFCs in a dose-dependent manner, which reached 9.70 ± 1.51 mm at the concentration of 4.0 μM (Figure 1A). This ticagrelor effect was significantly reduced in the presence of ADA (7.12 ± 0.79 mm compared to 9.70 ± 1.51 mm, *p* < 0.03) (Figure 2A). Furthermore, adenosine significantly increased tube formation by ECFCs in a dose-dependent manner, reaching the 12.92 ± 2.57 mm at a concentration of 20 μM (Figure 1A). This adenosine effect was markedly reduced in the presence of ADA (6.98 ± 0.62 mm as compared to 12.92 ± 2.57 mm, *p* < 0.02) (Figure 2A). Based on the results of these experiments, we next studied the effects of the combination of 1.0 μM ticagrelor with 5 μM adenosine on tube formation by ECFCs. As shown in Figure 1A, the effect of this combination was significantly higher compared to those of the same concentrations of either ticagrelor or adenosine. This effect was also significantly reduced in the presence of ADA (8.21 ± 1.81 mm compared to 17.02 ± 2.59 mm, *p* < 0.005) (Figure 2A). VEGF, used as an agonist in the positive control experiments, induced a dose-dependent increase in tube formation by ECFCs, reaching 14.56 ± 2.34 mm at the concentration of 25 ng/mL (Figure 1A). Similar results were obtained when HUVECs were used instead of ECFCs (Figure 1B).

Moreover, the spheroid sprout formation by ECFCs in the presence of 0.1% *w*/*v* BSA/PBS was 0.41 ± 0.08 mm, similar to that formed in the presence of 0.4% *v*/*v* DMSO (Figure 1C). Ticagrelor significantly increased the spheroid sprout formation by ECFCs in a dose-dependent manner, reaching 1.50 ± 0.40 mm at the concentration of 4.0 μM (Figure 1C). This effect was significantly reduced in the presence of ADA (0.63 ± 0.07 mm compared to 1.50 ± 0.40 mm, *p* < 0.02) (Figure 2B). Adenosine also significantly increased the sprout formation by ECFCs in a dose-dependent manner, reaching 1.85 ± 0.40 mm at the concentration of 20 μM (Figure 1C). This adenosine effect was significantly reduced in the presence of ADA (0.52 ± 0.06 mm compared to 1.85 ± 0.40 mm, *p* < 0.01) (Figure 2B). The combination of 1.0 μM ticagrelor with 5 μM adenosine also induced the sprouting of ECFCs, which was significantly higher as compared to that observed in the presence of ticagrelor or adenosine alone at the same concentrations (Figure 1C). This effect was also significantly reduced in the presence of ADA (0.75 ± 0.03 mm compared to 2.00 ± 0.06 mm, *p* < 0.005) (Figure 2B).VEGF, used as an agonist in positive control experiments, induced a dose-dependent increase in the spheroid sprout formation by ECFCs reaching the 2.00 ± 0.40 mm at the concentration of 25 ng/mL (Figure 1C).

**Figure 3:**
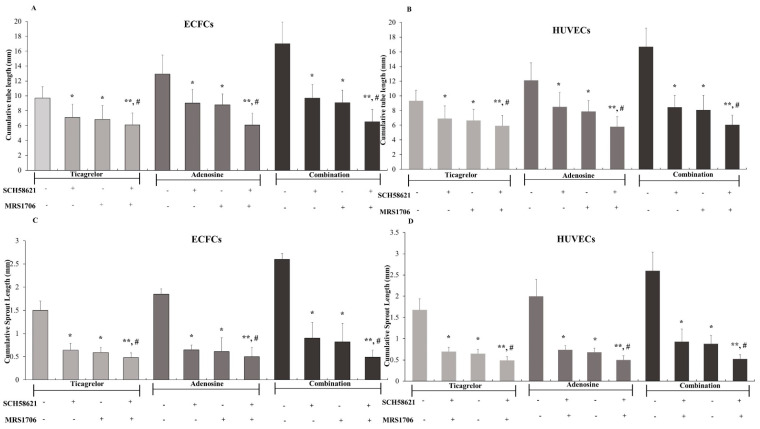
The effects of the adenosine receptor A_2A_ antagonist SCH58621, adenosine receptor A_2B_ inverse agonist, and antagonist MRS1706 on capillary-like tube formation by (**A**) ECFCs and (**B**) HUVECs, and on spheroid sprout formation by (**C**) ECFCs and (**D**) HUVECs, induced by ticagrelor, adenosine, and their combination. The values represent the means ± SDs from at least 6 independent biological replicates each performed in 2 technical replicates. * *p* < 0.02 and ** *p* < 0.01 compared to ticagrelor, adenosine, or their combination in the absence of SCH58621 and MRS1706. ^#^ *p* < 0.05 compared to ticagrelor, adenosine, or their combination in the presence of either SCH58621 or MRS1706. HUVECs: human umbilical vein endothelial cells, ECFCs: endothelial colony-forming cells.

**Figure 4:**
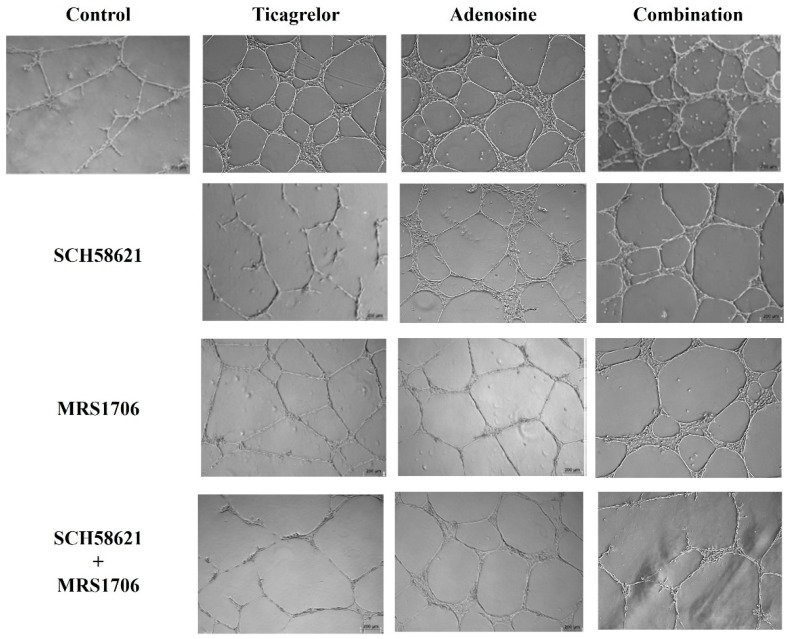
Representative images of 2D capillary-like tube formation by ECFCs that were treated with ticagrelor, adenosine, and their combination in the presence of adenosine receptor A_2A_ antagonist SCH58621, adenosine receptor A_2B_ inverse agonist, and antagonist MRS1706. Scale bar 100 μΜ.

After the preliminary experiments with a various concentration range of ticagrelor and adenosine regarding the cumulative length of the tube network and the spheroid sprout formation by ECFCs, we next performed experiments using the ticagrelor concentration of 4.0 μM, which induced the maximum of tube formation. Then we studied the effects of the AR antagonists, DPCPX (A_1_ antagonist), SCH58621 (A_2A_ antagonist), MRS1706 (A_2B_ inverse agonist and antagonist), and MRS1220 (A_3_ antagonist), on this ticagrelor effect. As shown in Figure 3A, SCH58621 and MRS1706 significantly reduced ticagrelor-induced tube formation, a phenomenon more pronounced when the combination of these compounds was used. By contrast, no inhibition was observed in the presence of the AR antagonists DPCPX and MRS1220, which, when combined with ticagrelor, demonstrated tube network cumulative lengths of 8.86 ± 0.23 mm and 9.30 ± 0.60 mm, respectively (Supplementary Figure S3A). These values were not significantly different compared to those observed in the presence of ticagrelor alone. SCH58621 and MRS1706, as well as their combination, significantly reduced the effects of 20 μM adenosine, as well as the effects of its combination with ticagrelor (Figure 3A). The cumulative length of the tube network observed in the presence of adenosine (12.92 ± 0.57 mm) was similar to those observed for the combination of adenosine with both DPCPX (11.58 ± 0.46 mm) and MRS1220 (12.30 ± 0.75 mm) (Supplementary Figure S3A). Similarly, these two AR antagonists did not affect the cumulative length of the tube network observed in the presence of the combination of ticagrelor with adenosine. Specifically, the cumulative length of the tube network observed in the presence of the combination of ticagrelor with adenosine (17.02 ± 0.59 mm) was equivalent to those observed for the combination of ticagrelor and adenosine with both DPCPX (16.58 ± 0.65 mm) and with MRS1220 (16.12 ± 0.13 mm). Representative images of the 2D capillary network formation assays for ECFCs treated with 4.0 μM ticagrelor, 20 μM adenosine, or their combination, in the presence of SCH58621 and MRS1706, are shown in Figure 4. Similar results were obtained when HUVECs were used instead of ECFCs (Figure 3B). 

**Figure 5:**
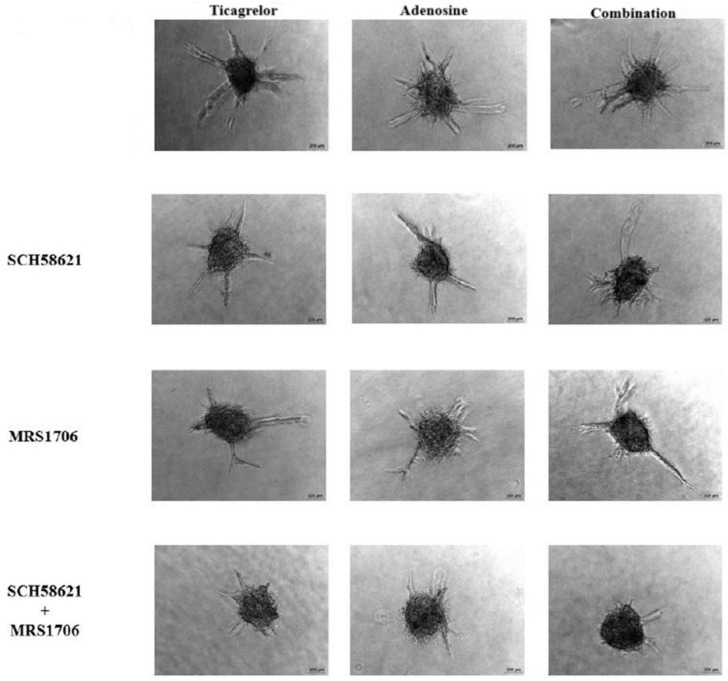
Representative images of 3D spheroid sprout formation by ECFCs that were treated with ticagrelor, adenosine, and their combination in the presence of adenosine receptor A_2A_ antagonist SCH58621, adenosine receptor A_2B_ inverse agonist, and antagonist MRS1706. Scale bar 200 μM.

By using the ticagrelor concentration of 4.0 μM, we then studied the possible effects of the AR antagonists SCH58621, MRS1706, DPCPX, and MRS1220 on the ticagrelor-induced sprouting of ECFCs. As shown in Figure 3C, SCH58621 or MRS1706 significantly reduced ticagrelor-induced sprout formation, a phenomenon more pronounced when the combination of these compounds was used. SCH58621, MRS1706, and their combination also significantly reduced the effects of 20 μM adenosine as well as the effects of the combination of ticagrelor with adenosine (Figure 3C). By contrast, no inhibition was observed in the presence of the AR antagonists DPCPX and MRS1220, which, when combined with ticagrelor, demonstrated 1.59 ± 0.18 mm and 1.54 ± 0.13 mm spheroid sprout formation by ECFCs, respectively (Supplementary Figure S3B). These values were not significantly different compared to those observed in the presence of ticagrelor alone. The spheroid sprout formation by ECFCs observed in the presence of adenosine (1.85 ± 0.09 mm) was similar to those observed for the combination of adenosine with both DPCPX (1.89 ± 0.37 mm) and MRS1220 (1.78 ± 0.26 mm) (Supplementary Figure S3A). Similarly, these two AR antagonists did not affect the spheroid sprout formation by ECFCs. Specifically, the spheroid sprout formation by ECFCs observed in the presence of the combination of ticagrelor with adenosine (2.00 ± 0.12 mm) was equivalent to those observed for the combination of ticagrelor and adenosine with both DPCPX (2.09 ± 0.23 mm) and MRS1220 (1.96 ± 0.26 mm) (Supplementary Figure S3A). Representative images of 3D spheroid sprout formation by ECFCs treated with ticagrelor and adenosine in the presence of SCH58621 and MRS1706 are shown in Figure 5. Similar results were obtained when HUVECs were used instead of ECFCs (Figure 3D). 

## 3. Discussion

The present study shows, for the first time, that ticagrelor and adenosine stimulate ECFCs and mature ECs and induce angiogenesis in vitro, as determined by two different assays—capillary-like tube and spheroid sprout formation. Importantly, our study further shows that the angiogenic effects of ticagrelor mimic those of adenosine since the action of both agents is mainly mediated through the A_2A_AR and A_2B_AR. 

Angiogenesis occurs through a convergence of diverse signaling mechanisms in which the autocrine effect of VEGF plays a prominent role [31]. In accordance with this, our results indicate that, in positive control experiments, VEGF is a potent agonist of angiogenesis in ECFCs and mature ECs. An important contribution to angiogenesis may be ARs, especially A_2A_AR and A_2B_AR [32,33,34,35]. In this regard, it has been demonstrated that A_2A_AR has pro-angiogenic properties and increases the tube formation in pulmonary ECs [36]. Furthermore, previous studies have reported that a selective A_2A_AR agonist (CGS21680) significantly enhances angiogenesis and wound repair only in wild-type and not in A_2A_AR knockout mice [37]. Other studies have also supported the central role of A_2B_AR in angiogenesis by increasing the expression levels of angiogenic factors, including VEGF and interleukin-8 in microvascular ECs [38], as well as by regulating the expression of other angiogenic factors, including endothelial nitric oxide synthase (eNOS) in ECs [28]. In addition, it has been shown that the stimulatory effect of the adenosine analogue 5′-(N-ethylcarboxamido)adenosine (NECA) on angiogenesis is inhibited by MRS1706 (an A_2B_AR inverse agonist and antagonist) in zebrafish embryos [39]. Our results are in agreement with these published data, supporting the pivotal role and stimulatory effect of A_2A_AR and A_2B_AR in angiogenesis by mature ECs. Our results further show that these two receptors also play an important role in angiogenesis by ECFCs, the type of EPC that displays an increased ability to form capillary tube structures [8,11]. 

The present study further shows that the angiogenic effects of ticagrelor on capillary-like tube and spheroid sprout formation by ECFCs and mature ECs is inhibited by the A_2A_AR and A_2B_AR antagonists, thus suggesting that an important role in this action is played by adenosine. This is further supported by the finding that the adenosine angiogenic effects in both assays were significantly increased in the presence of ticagrelor. Therefore, one possible underlying mechanism for this ticagrelor action is through the ENT-1 inhibition, thus preventing the reduction of adenosine levels through this nucleoside transporter and, therefore, increasing its half-life [21,22,23]. In this regard, several studies have suggested that ticagrelor may affect the EC function through an adenosine-mediated, P2Y_12_-independent mechanism of action [15,16,17,18,40]. However, other studies have demonstrated that the increase in the half-life of adenosine may not represent the only mechanism by which ticagrelor improves EC function, suggesting that other molecular targets may be involved. [41,42,43,44]. In this regard, it has been shown that ticagrelor reduces circulating epidermal growth factor levels, which positively affects EC function by increasing eNOS activity [18]. Furthermore, ticagrelor negatively regulates the NF-ΚB signaling pathway in ECs via inhibiting the degradation of IKBα and the phosphorylation and entry into the nucleus of p65 to alleviate cellular dysfunction [45]. Concerning the possibility that ticagrelor affects ECs through its main mechanism of action, i.e., through the inhibition of the P2Y12 receptor, we should mention that there is a paucity of data on the possible existence of this receptor on ECFCs, whereas there are contradictory data on the expression of functional receptors in mature ECs [46,47,48,49]. Very recently, we demonstrated that in a coculture of platelets with outgrowth endothelial cells (OECs), ticagrelor could promote platelet–OEC interaction and OEC functionality indirectly by inhibiting ADP-induced platelet aggregation [50]. Consequently, the effects of ticagrelor on EC and EPC functionality, particularly on their angiogenic properties, which are the subject of the present study, should be further investigated. The limitation of the present study was the use of HUVECs as a secondary cell population since they are primary cells and only viable for a limited time. Like other cells in culture, they change their expression profile and, therefore, their phenotype and behavior over time and through repeated passage events. However, subsequent studies will include microvascular endothelial cells or artery endothelial cells since angiogenesis mainly occurs inside the vessels. Moreover, the lack of proteomics and genomics analyses to prove that ticagrelor works via the adenosine receptors A2A and A2B leads to some mechanistic ambiguity; this reflects a critical area for further investigation. While our study primarily focused on the adenosine pathway due to its well-documented role in angiogenesis and its pharmacological relevance to ticagrelor’s mode of action, we agree that the possibility of additional molecular targets warrants exploration. Future studies could employ omics-based approaches, such as proteomics or transcriptomics, to identify alternative pathways or mediators involved in ticagrelor’s angiogenic activity. Such efforts will provide a more comprehensive understanding of its mechanistic landscape and therapeutic potential.

In conclusion, our data demonstrate that ticagrelor exerts angiogenic actions and induces the integration of EPCs and mature ECs into vascular structures. An important role in this effect may be played by adenosine acting through its receptors, A_2A_ and A_2B_. The significance of these results at the clinical level in patients with atherothrombotic events and treated with this antiplatelet agent needs to be investigated. 

## 4. Material and Methods

### 4.1. Materials

Ticagrelor was kindly provided by AstraZeneca (Gothenburg, Sweden) and dissolved in dimethyl sulfoxide (DMSO) purchased from Sigma-Aldrich (Darmstadt, Germany) to prepare a 10 mM stock solution. Adenosine, DPCPX (A_1_AR antagonist), 1,1’-dioctadecyl-3,3,3’,3’-tetramethylindocarbocyanine-labeled acetylated LDL (DiI-ac-LDL), and adenosine deaminase were purchased from Sigma-Aldrich (St. Louis, MO, USA), and MRS1220 (A_3_AR antagonist), SCH58621 (A_2A_AR antagonist), and MRS1706 (A_2B_AR inverse agonist and antagonist) were purchased from Tocris Bioscience (Bristol, UK). EGM-2 BulletKit and EGM-Plus BulletKit, as well as passage 1 human umbilical vein endothelial cells (HUVECs), were purchased from Lonza (Walkersville, MD, USA). The Human Cluster of Differentiation 34 (CD34) MicroBead Kit and fetal bovine serum (FBS) were purchased from Miltenyi Biotec (Bergisch Gladbach, Germany) and Gibco Life Technologies (Karlsruhe, Germany), respectively. Biocoll separating solution was purchased from Biochrom AG (Berlin, Germany). Anti-human VEGFR2/KDR (kinase insert domain receptor)-phycoerythrin (PE) monoclonal antibody was purchased from R&D Systems (Minneapolis, MN, USA), and anti-human CD34-fluorescein isothiocyanate (FITC), CD45-PE, cluster of differentiation 31 (CD31)-PE monoclonal antibodies, and Matrigel Growth Factor Reduced Basement Membrane Matrix were purchased from BD Biosciences (San José, CA, USA). VEGF was purchased from PeproTech (Rocky Hill, NJ, USA). FITC-conjugated Ulex europaeous agglutinin (UEA)-1 lectin, rabbit anti-human von Willebrand factor (vWF), and Alexa Fluor^®^ goat anti-rabbit secondary antibody vWF were purchased from Biomedical Technologies (Stoughton, MA, USA), Dako (Carpinteria, CA, USA), and Invitrogen (Carlsbad, CA, USA), respectively.

### 4.2. Isolation of CD34^+^ Progenitor Cells and Differentiation into ECFCs

CD34^+^ progenitor cells were kindly supplied by the Department of Obstetrics and Gynecology, School of Medicine, University of Ioannina. The CD34^+^ progenitor cells were isolated from mononuclear cells using the human CD34 MicroBead Kit following the manufacturer’s instructions and protocol [30]. Briefly, cord blood was collected into sterilized tubes containing 20 mM EDTA/PBS solution as an anticoagulant, and the mononuclear cells were separated by density gradient centrifugation on Biocoll separation solution at 400× *g* and 20 °C for 35 min. The CD34^+^ cells were cultured in EGM-2 BulletKit supplemented with 10% FBS on collagen-coated cell-culture plates at 37 °C and 5% CO_2_ for 20–30 days. The medium was changed daily for the first 7–14 days and every 2 days afterward until the appearance of EC colonies, which were further cultured until the formation of a confluent monolayer of ECFCs. The ECFCs were characterized as we previously described [30]. All experiments of the present work were performed using the ECFCs of passage 3. More specifically, the cells were cultured with EGM-Plus BulletKit supplemented with 10% FBS at 37 °C and 5% CO_2_ on collagen-coated dishes, until reaching 70–90% confluency.

### 4.3. Culture of HUVECs

The passage 1 HUVECs were cultured in EGM-Plus BulletKit supplemented with 10% FBS at 37 °C and 5% CO_2_. After colonies were formed, the cells were detached and cultured in collagen-coated dishes, until reaching 70–90% confluency in passage 2. All experiments of the present work were performed using the HUVECs of passage 2 as control cells.

### 4.4. Angiogenesis Assays

Angiogenesis studies were performed in vitro using 2 different assays, the capillary-like tube formation and the spheroid-based angiogenesis assays. In both assays, ticagrelor was used at three different concentrations of 1.0, 2.0, and 4.0 μM, and adenosine was studied at the concentrations of 5, 10, and 20 μM. In some experiments, the combination of 1.0 μM ticagrelor with 5 μM adenosine was studied. Some of the above experiments were also performed in the presence/absence of 3 U/mL ADA added to cell culture for 5min prior to the addition of ticagrelor, adenosine, or their combination. In other experiments, the possible effects of DPCPX (A_1_AR antagonist), SCH58621 (A_2A_AR antagonist), MRS1706 (A_2B_AR inverse agonist and antagonist), and MRS1220 (A_3_AR antagonist) on both angiogenesis assays were also studied. Positive control experiments were performed using vascular endothelial growth factor (VEGF) as an angiogenesis activator using three different concentrations (5.0, 25.0, and 50.0 μM). A solution of BSA/PBS, 0.1% *w*/*v* was used as the vehicle for VEGF, while DMSO (at a final concentration of 0.4% (*v*/*v*) was used as a vehicle of the tested substances (ticagrelor, adenosine, and AR antagonists). 

### 4.5. Capillary-Like Tube Formation Assay

An EC tube formation assay was performed following previously described experimental methodology [51]. Briefly, confluent ECFCs and HUVECs were detached by treatment with trypsin/EDTA, and 2 × 10^5^ cells of each cell type were seeded in 12-well cell-culture plates coated with 200 μL Matrigel Growth Factor Reduced Basement Membrane Matrix. The cells were incubated with ticagrelor, adenosine, or their combination or with VEGF for 16 h. The cells were also incubated with 10 μM SCH58621 [52], 5 μM MRS1706 [53], or their combination, as well as with 30 μM DPCPX [54] or 10 μM MRS1220 [55] for 5 min, prior to the addition of ticagrelor, adenosine, or their combination. The tube network formation was quantified after 16 h in culture at 37 °C and 5% CO_2_ by measuring the cumulative tube length in five random microscopic fields with a computer-assisted Axiovert 100 M inverted microscope equipped with a Plan-NEOFLUAR objective (5×/0.30; Carl Zeiss, Jena, Germany). The images were analyzed by using Axiovision 4.9.1 software (Carl Zeiss, Jena, Germany). Microscopic images were taken by two independent experts who were blinded to the test conditions.

### 4.6. Spheroid-Based Angiogenesis Assay

EC spheroids of a defined cell number (10^5^ cells) were generated as previously described [51]. Briefly, confluent ECFCs and HUVECs were incubated overnight in hanging drops to form spheroids and then embedded in collagen and incubated with ticagrelor, adenosine, or their combination, or with VEGF for 24 h to induce sprouting. Cell spheroids were also incubated with 10 μM SCH58621, 5 μM MRS1706, or their combination, as well as with 30 μM DPCPX or 10 μM MRS1220 for 5 min prior to the addition of ticagrelor, adenosine, or their combination. Sprouting was quantified by taking micrographic images using an Axiocam MR digital camera and an Axiovert 100 M inverted microscope with a Plan-NEOFLUAR objective (20×/0.30; Carl Zeiss, Jena, Germany). The cumulative length of all sprouts grown out of each spheroid was measured using Axiovision 4.9.1 software (Carl Zeiss, Jena, Germany), analyzing 10 spheroids per group in each experiment. Microscopic images were taken by two independent experts who were blinded to the test conditions.

### 4.7. Statistical Analysis

The values are expressed as the means ± standard deviation (SDs). Due to the small sample size, pairwise comparisons were tested for significance with a two-tailed nonparametric Mann–Whitney *U* test or Kruskal–Wallis 1-way analysis of variance (ANOVA). Statistical significance was defined as *p* < 0.05. No statistical method was used to predetermine the sample size. No samples were excluded from the analysis. The experiments were not randomized. Moreover, due to the pairwise comparisons across different concentrations and receptor antagonists, we also performed Bonferroni’s post hoc analysis. Statistical analysis of the data was performed using Statistical Package for the Social Sciences (SPSS) v23 (Chicago, IL, USA).

## Data Availability

All data are located at the Atherothrombosis Research Center and will be available upon request from the corresponding author Professor Alexandros D. Tselepis (atselep@uoi.gr).

## Supplementary Materials

The following supporting information can be downloaded at: https://www.mdpi.com/article/10.3390/ijms252413343/s1.

## Abbreviations

Adenosine diphosphate	ADP
Adenosine receptors	ARs
Dimethyl sulfoxide	DMSO
Endothelial colony-forming cells	ECFCs
Endothelial progenitor cells	EPCs
Equilibrative nucleoside transporter-1	ENT-1
Fetal bovine serum	FBS
Human umbilical vein endothelial cells	HUVECs
Outgrowth endothelial cells	OECs
Vascular endothelial growth factor	VEGF
von Willebrand factor	vWF
