# Supplementary material for: Ticagrelor Induces Angiogenesis in Progenitor and Mature Endothelial Cells In Vitro: Investigation of the Possible Role of Adenosine
Source: Int J Mol Sci. 2024 Dec 12;25(24):13343. doi: 10.3390/ijms252413343 (PMC11727715; doi:10.3390/ijms252413343)
Supplement: Supplementary file 1 [file ijms-25-13343-s001.zip › ijms-3262080-supplementary.pdf]

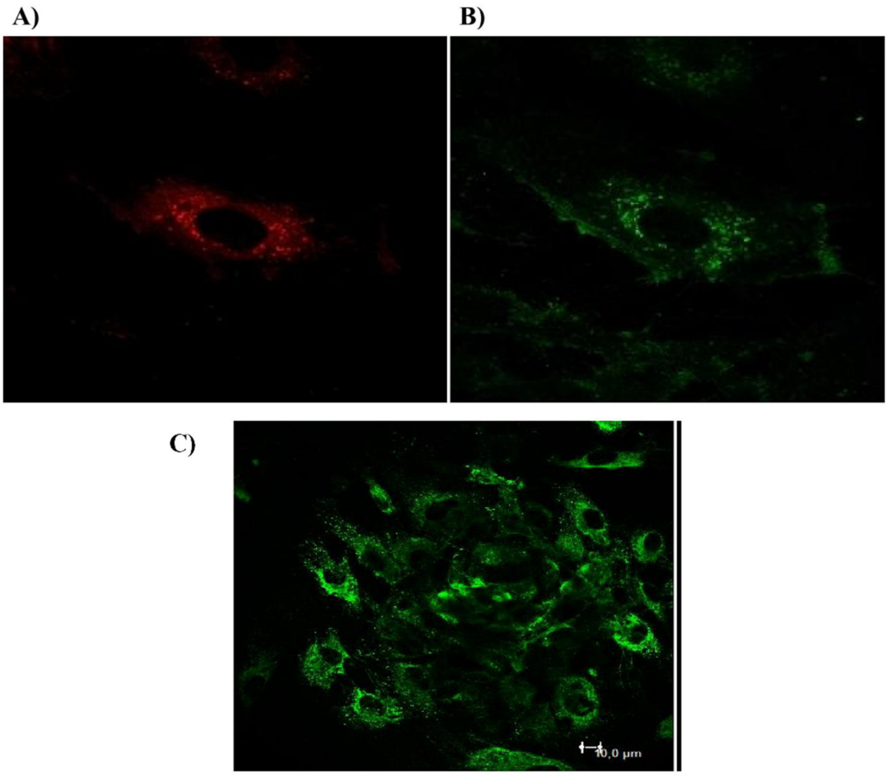

Supplementary Figure S1: Representative fluorescence images of ECFCs stained with **A.** DiI-ac-LDL **B.** FITC- (UEA)-1 lectin and **C.** vWF.

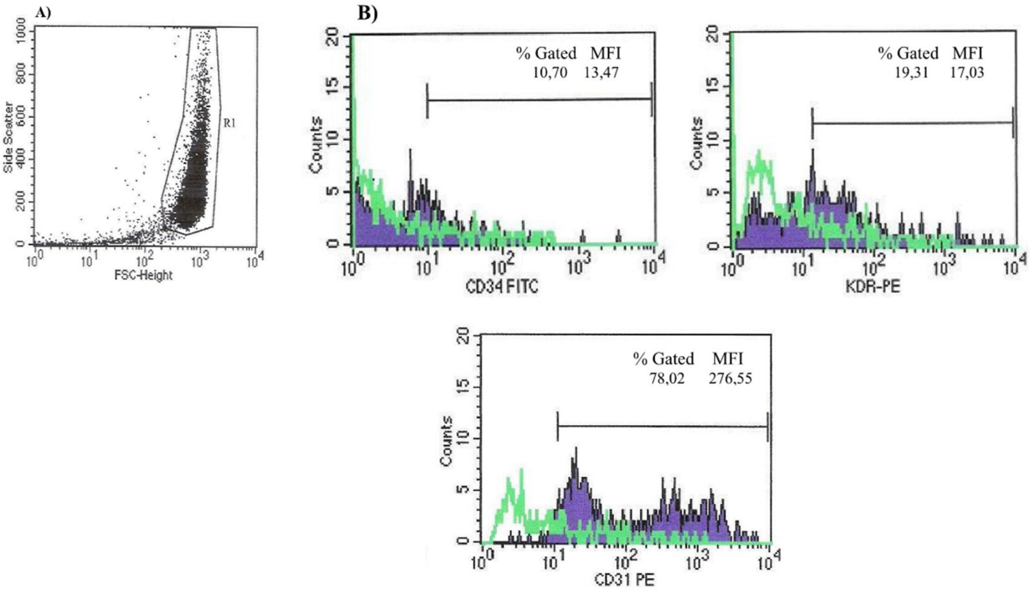

Supplementary Figure S2: A. The cytometric profile of the isolated ECFCs. **B**. The expression of CD34-FITC, KDR-PE and CD31-PE of the isolated ECFCs.

A.

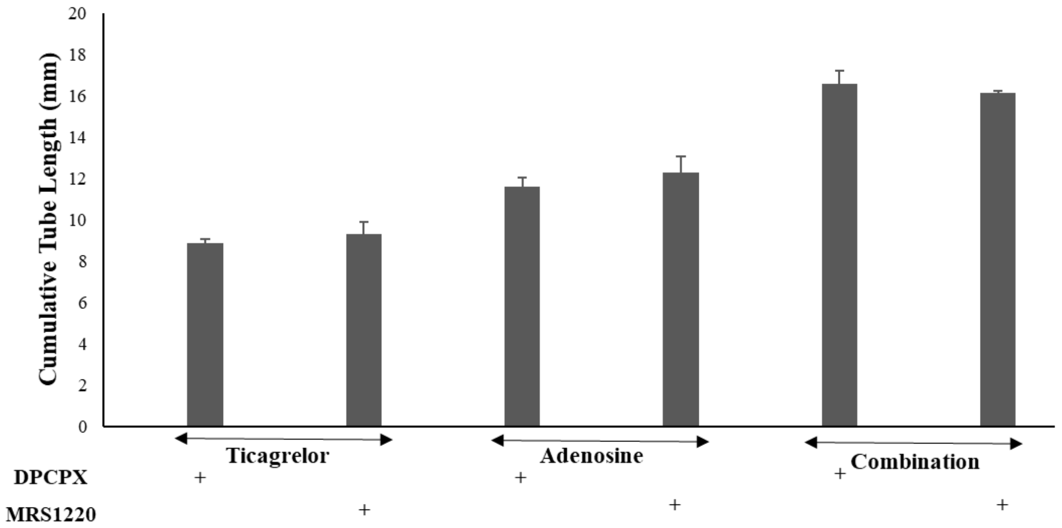

B.

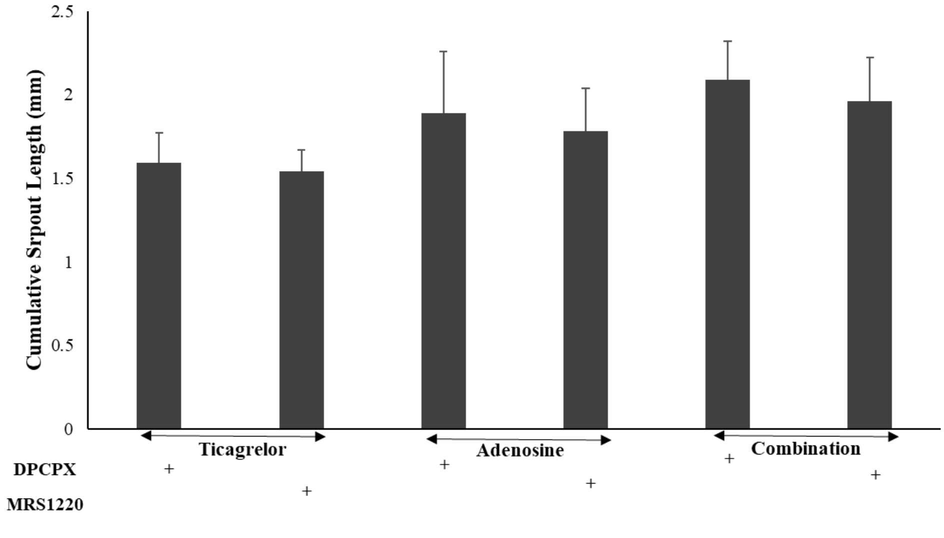

Supplementary Figure S3: A. Effect of the adenosine receptors DPCPX and MRS1220 on the effect of ticagrelor, adenosine or their combination on capillary-like tube formation by **B**. Effect of the adenosine receptors DPCPX and MRS1220 on the effect of ticagrelor, adenosine or their combination on spheroid sprout formation.
